# Confirmatory and Exploratory Factor Analysis for Validating the Phlegm Pattern Questionnaire for Healthy Subjects

**DOI:** 10.1155/2016/2696019

**Published:** 2016-03-09

**Authors:** Hyunho Kim, Boncho Ku, Jong Yeol Kim, Young-Jae Park, Young-Bae Park

**Affiliations:** ^1^Department of Biofunctional Medicine and Diagnostics, College of Korean Medicine, Kyung Hee University, Seoul 02447, Republic of Korea; ^2^Korean Medicine Fundamental Research Division, Korea Institute of Oriental Medicine, Daejeon 34054, Republic of Korea

## Abstract

*Background*. Phlegm pattern questionnaire (PPQ) was developed to evaluate and diagnose phlegm pattern in Korean Medicine and Traditional Chinese Medicine, but it was based on a dataset from patients who visited the hospital to consult with a clinician regarding their health without any strict exclusion or inclusion. In this study, we reinvestigated the construct validity of PPQ with a new dataset and confirmed the feasibility of applying it to a healthy population.* Methods*. 286 healthy subjects were finally included and their responses to PPQ were acquired. Confirmatory factor analysis (CFA) was conducted and the model fit was discussed. We extracted a new factor structure by exploratory factor analysis (EFA) and compared the two factor structures.* Results*. In CFA results, the model fit indices are acceptable (RMSEA = 0.074) or slightly less than the good fit values (CFI = 0.839, TLI = 0.860). Many average variances extracted were smaller than the correlation coefficients of the factors, which shows the somewhat insufficient discriminant validity.* Conclusions*. Through the results from CFA and EFA, this study shows clinically acceptable model fits and suggests the feasibility of applying PPQ to a healthy population with relatively good construct validity and internal consistency.

## 1. Background

In Korean Medicine (KM) and Traditional Chinese Medicine (TCM), pathologic pattern identification is a very important diagnostic tool for KM or TCM doctors to evaluate a patient's health status and to decide clinical interventions. Pattern, which is a subcategory of a disease or a disorder, is defined as a diagnostic conclusion based on the pathological changes closely observed and holistically analyzed and may include a variety of information such as causes, locations, and properties of disorders or diseases [1, 2]. To identify a pattern, various kinds of clinical information are needed, and they are acquired by four examinations: inspection, listening and smelling, inquiry, and palpation. However, objective and quantitative tools are essential because intertester reliability cannot be guaranteed due to the subjective aspects of the examinations. Moreover, because the inquiry is an indirect method to acquire clinical information from the patients, inquiry lists or questionnaire items should be developed carefully.

Questionnaires based on the survey methodology are convenient and useful for the measurement and evaluation of subjective concepts or personal feelings and are therefore broadly used throughout various research fields such as psychology, medicine, education, sociology, and marketing. To support KM or TCM clinicians with fast and quantitative analysis of important signs and symptoms in the pattern identification, several KM pattern identification questionnaires were developed and validated [3–7]. Among these, the phlegm pattern questionnaire (PPQ) was developed to evaluate and diagnose the phlegm status of a subject. KM and TCM have broadly defined phlegm concept and narrowly defined phlegm concept. The former induce various signs and symptoms such as dizziness or palpation, which resulted from the internal disruption of the body fluid metabolism. The latter is visible phlegm such as nasal discharge or sputum, mainly from the lungs and the upper respiratory tract [4]. According to the KM and TCM theories, phlegm patterns have a wide spectrum of clinical signs and symptoms and often combine with other pathogenic patterns to form more complex patterns. According to Zhu Zhenheng, a famous TCM physician in Yuan dynasty, nine out of ten diseases are associated with phlegm. Therefore, phlegm pattern has important clinical value in diagnosing many diseases and identifying patterns in KM and TCM.

In general, the development and validation study of a questionnaire are conducted at the same time, but additional validation studies are needed to apply the questionnaire to another population. Because the factor structure is easily influenced by sampled data, repetitive revalidation studies are needed to overcome the sampling bias and to confirm the latent variable structure. PPQ was validated with the limited dataset obtained during development [4], and there has been no revalidation clinical study on a similar or different population. Therefore, the aims of this study were to investigate the factor structure of PPQ from [4] with a new dataset of healthy people, to figure out the new structure of latent variables, and to discuss its validation and applicability to a healthy population.

## 2. Methods

### 2.1. Participants and Criteria

**Figure 1:**
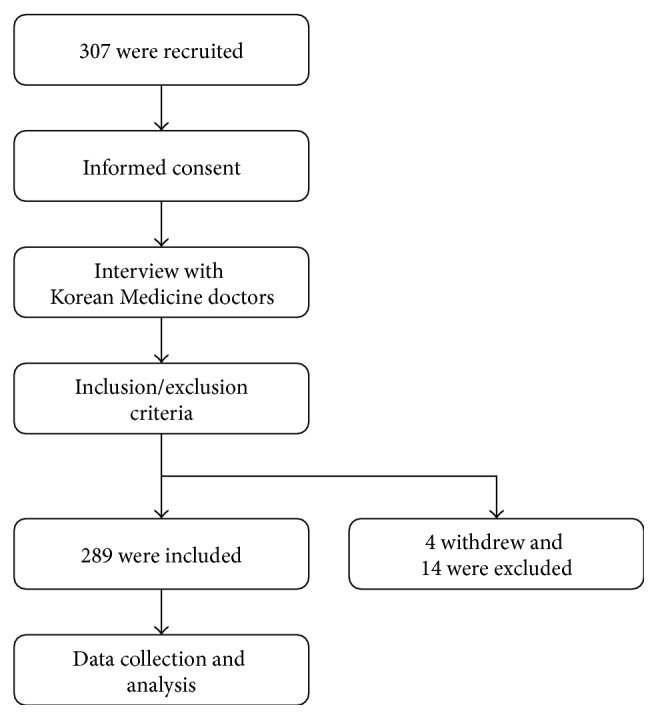
Clinical study procedure.

We recruited healthy volunteers in their 20s, 30s, and 40s from two sites in Korea: Kyung Hee University Korean Medicine Hospital in Seoul and Cheonan Oriental Hospital of Daejeon University in Cheonan. Korean Medicine doctors minutely interviewed the 307 volunteers (107 in Seoul and 200 in Cheonan) and included or excluded them according to the inclusion and exclusion criteria of this clinical study. Basically, those who can communicate with clinical research coordinators about their health status were included and then excluded with the following exclusion criteria: medical operation or procedure during one-month-long period prior to interview; excessive control of diet for any clinical treatment or weight reduction; pregnancy or breast-feeding; medication due to any diagnosed disease; severe pain or discomfort from which diseases are suspected. Fourteen participants were excluded according to the exclusion criteria, and four participants withdrew from participation (Figure 1). Finally, data from 289 participants were analyzed, and their basic sociodemographic characteristics are shown in Table 1.

This study design and ethics were approved by the institutional review board of Kyung Hee University Korean Medicine Hospital (KOMCIRB-2014-70) and Cheonan Oriental Hospital of Daejeon University (M2014-01-1). Informed consent was obtained from all individual participants included in the study.

### 2.2. Phlegm Pattern Questionnaire

The self-rated phlegm pattern questionnaire (PPQ) was developed by Delphi method based on the clinical expert opinions and the contexts of Korean Medicine and Traditional Chinese Medicine. It was revised and validated by various survey methodologies including exploratory factor analysis [4]. The PPQ consists of 25 items and the original form is presented in the Appendix. Each item is rated on a 7-point Likert scale: 1 = disagree very strongly; 2 = disagree strongly; 3 = disagree; 4 = neither agree nor disagree; 5 = agree; 6 = agree strongly; 7 = agree very strongly. Internal consistency of PPQ, which was examined by Cronbach's *α*, is 0.919, and the proposed optimum cut-off score is five when the 7-point Likert scale is dichotomized where 5, 6, and 7 points are coded as 1, and others are coded as 0.

### 2.3. Analysis Procedure

First, in order to investigate whether the factor structure can be replicated in the new dataset from 289 participants, confirmatory factor analysis (CFA) was conducted. Several model fit indices and their criteria were used to examine the goodness-of-fit of the model with the given dataset: goodness-of-fit index (GFI), adjusted goodness-of-fit index (AGFI), normed fit index (NFI), Tucker-Lewis Index (TLI), comparative fit index (CFI), and root mean square error of approximation (RMSEA). After evaluating the model fit, we calculated construct reliability (CR) for convergent validity and average variance extracted (AVE) for discriminant validity. Second, after performing the CFA, we extracted a more suitable factor structure from the new dataset. We then performed exploratory factor analysis (EFA) with maximum likelihood factoring. Maximum likelihood and principal axis factoring are generally recommended extraction methods [8]. Extracted factors were rotated by varimax rotation. Finally, the reliability of items in each factor was examined by Cronbach's *α*.

We used AMOS (SPSS Inc., Chicago, IL, USA) for CFA, SPSS Statistics 19 (SPSS Inc., Chicago, IL, USA) for EFA, and Microsoft Office Excel 2010 (Microsoft, Redmond, WA, USA) for other calculations.

## 3. Results and Discussion

### 3.1. Confirmatory Factor Analysis

**Figure 2:**
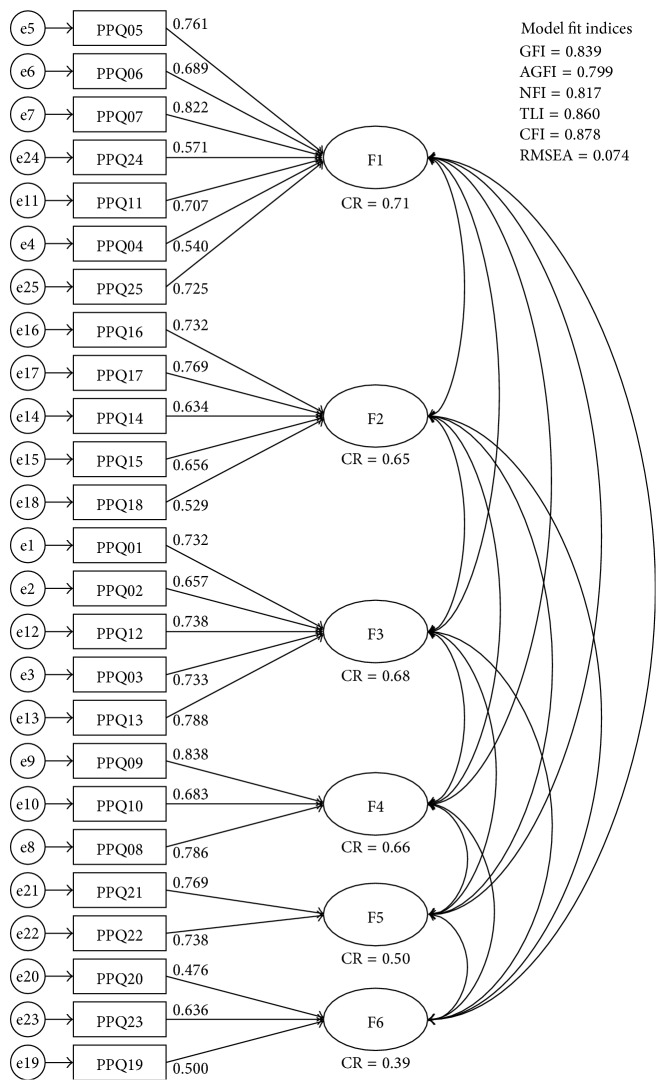
Results of the confirmatory factor analysis of the PPQ for healthy subjects.

Six-factor model from a previous study [4] for CFA is presented in Figure 2. Item numbers (PPQxx) in the figure correspond with the item numbers in the Appendix. Factor loadings and CRs for convergent validity are also presented in Figure 1. The model fit indices were as follows: GFI = 0.839, AGFI = 0.799, NFI = 0.817, TLI = 0.860, CFI = 0.878, and RMSEA = 0.074. Discriminant validity of this model can be examined by the correlation coefficients and the AVE in Table 2.

### 3.2. Exploratory Factor Analysis

Before EFA, the Kaiser-Meyer-Olkin (KMO) test and Bartlett's test of sphericity were conducted to evaluate the factorability. The KMO measure of sampling adequacy was 0.922 and the significance of Bartlett's test of sphericity was less than 0.001, meaning that EFA can be applied to the obtained dataset [9].

EFA was conducted with the obtained data to extract the new factor structure and to examine the construct validity. Factors were extracted by the maximum likelihood method and rotated by varimax rotation. The number of factors was decided in consideration of the scree-plot, cumulative variance explained, interpretability, and Kaiser's criterion. A total of five factors were extracted and rotated, and the cumulative variance explained was 51.81%. Items (20), (23), and (19) have factor loading of less than 0.4 for all factors. Factor structures of the EFA results and the previous model from Park's study are compared in Table 4.

### 3.3. Internal Consistency

One of the most popular estimates of internal consistency is Cronbach's *α*. Factor Cronbach's *α* and the item-delete Cronbach's *α* of each item are presented in Table 5. Generally, if *α* ≥ 0.9, the internal consistency is considered to be excellent, and if 0.7 ≤ *α* < 0.9, it is considered to be good. All the extracted factors have good internal consistency. According to the analysis results, if items (20), (19), and (15) are deleted, Cronbach's *α* of the corresponding factor increases slightly.

## 4. Discussion

In this study, we investigated whether a new dataset from healthy subjects is suitable for the 6-factor model devised in a previous validation study [4]. For that, CFA was conducted and model fits were discussed. Next, EFA was conducted to extract the new factor structure from the dataset and compare it with the 6-factor model. Data were obtained in the clinical trial with strict inclusion and exclusion criteria. Subjects are well distributed in their sex, age, and marital status (Table 1). The education level category was weighted towards high school due to the fact that many of the subjects are university students.

To discuss the model fit of CFA, we should consider the criteria of the various model fit indices. It has been suggested that RMSEA values less than 0.05 are good, values between 0.05 and 0.08 are acceptable, values between 0.08 and 0.1 are marginal, and values greater than 0.1 are poor [8]. Therefore, the RMSEA value of 0.074 in this sample indicates an acceptable fit. The GFI value of this sample, 0.84, is below 0.9, but the GFI and AGFI are known to depend on the sample size [10]. The CFI value is close to 0.9, which shows a relatively good fit [11]. The other fit indices, NFI and TLI, should be over 0.9 for a good fit [11], but in this sample, the two indices are a little below the criteria. Based on these indices, this sample has an acceptable fit to the 6-factor model.

In general, factor loadings and CR should be equal to or greater than 0.707 for good convergent validity [12]. From the CFA result of this study, fourteen loadings are greater than 0.707 and six loadings are between 0.6 and 0.707. Five loadings (those of items (19), (20), (18), (24), and (4)) are under 0.6. All items of factor 6 showed relatively low convergent validity. CR of factor 6 also has a low evaluation. Low convergent validity means the items have information of other factors rather than the corresponding factor alone. For good discriminant validity, AVE of one factor should be larger than any correlation coefficients between the factor and another one [12, 13]. If any factor has smaller AVE than correlation coefficients, it means the factors are correlated and that they do not measure well-separated latent concepts; however many correlation coefficients are larger than the corresponding AVEs in Table 2. Therefore, in this model and dataset, the factors are associated with one another. Two explanations are possible. First, latent factors that compose one concept in the real world cannot be absolutely independent. Additionally, because PPQ measures the pathologic phlegm pattern of KM or TCM, the factors of closely associated signs and symptoms can be expressed together. Second, because the subjects are healthy people, it is possible that the distinguishing signs and symptoms of a specific disease other than phlegm pattern were not expressed.

We conducted EFA to extract the new factor structure of the dataset and found a 5-factor structure model (Table 3). Items (20), (23), and (19) have all factor loadings of below 0.4. In fact, item (23) can be thought to have marginal factor loading, 0.39, but the other two items have values of equal to or less than 0.3. Thus, items (19) and (20) may influence the independency of the factors, and this is in agreement with the factor 6 result of the CFA in Figure 2. In comparison with Park's previous study, the dataset obtained in this trial has a more well-separated 5-factor structure. This is because the items of the 5-factor model have greater loadings for their corresponding factor and almost all items can be explained by one factor. However, it is a shortcoming of factor 5 that it has only two items, since according to the guidelines one factor should have more than two items if possible [14, 15].

Factor comparison of the 5- and 6-factor model is shown in Table 4, which shows the similarity of the factor structure. Items of “fatigue” and “feeling heavy” were combined with head-related signs and symptoms from the previous 6-factor model, but with dermatological signs and symptoms in this 5-factor model. Respiratory signs and symptoms, factor 5 in the 6-factor model and factor 3 in the 5-factor model, are almost identical in the two structures. Items of factor 6 in the 6-factor model—items (19), (20), and (23)—had the least variance explained. In this 5-factor model, they are scattered and bundled with factors 3, 1, and 2, respectively. Moreover, all loadings of the three items are below 0.4. In consideration of the CFA result (Figure 2) and the internal consistency evaluation (Table 5), the three items may be revaluated in a further revision or revalidation study. The other items showed acceptable or good internal consistency with high Cronbach's *α* (Table 5).

EFA is known as a data-driven method, and CFA as a theory-driven method. So the usage of EFA or CFA should be strictly considered and chosen according to the aim of a study, and aimless application of EFA and CFA to the same dataset should be avoided [16]. One can explore the latent variable structure of a dataset with EFA. On the other hand, CFA requires an* a priori* hypothesis or previous “theory” as CFA is a hypothesis testing method which tests whether the obtained dataset is suitable for a model [16]. Thus, in this study, we used CFA to discuss the model fit of the dataset obtained from the healthy subjects in the clinical trial to the previously extracted PPQ 6-factor structure. Also, we used EFA to extract the new factor structure according to the above-mentioned guidelines. Different from this study, Park's model was constructed with a dataset from patients who visited the hospital to consult with a clinician regarding their health without any strict exclusion or inclusion criterion [4]. Thus it was possible for patients, subhealthy subjects, and healthy subjects to participate in that study. This difference may have resulted in a small difference in factor structure.

In spite of our confirmation of the similar structure of the two models and a few items with relatively low reliability and validity in the models, this is still an exploratory study based on the survey research method and data-driven aspects. To overcome these limitations and to acquire the predictability and validity, prospective clinical trials should be carried out with the gold standard of pattern identification like agreement of several clinicians. If so, factor structures of patient group and healthy group can be compared and diagnostic value of the questionnaire can be discussed more.

## 5. Conclusion

A revalidation study of PPQ was conducted. A sample dataset obtained from clinical trial under strict conditions did not show the excellent model fit of the previous PPQ model, but the additional EFA indicated similar factor structures exist and it was hypothesized that the difference might come from a few items. In conclusion, PPQ can be applied to a healthy population with good construct validity and internal consistency for evaluating phlegm pattern, and it can be more improved if a few items are adjusted with further studies.

## Figures and Tables

**Table 1:** Sociodemographic characteristics of participants (*N* = 289).

Participants characteristics	Frequency (%) or mean (±SD)
Gender:	
Male	139 (48.1)
Female	150 (51.9)
Age (years):	35.94 (±8.46)
Twenties	79 (27.3)
Thirties	103 (35.6)
Forties	107 (37.0)
Marital status:	
Unmarried	137 (47.4)
Married	141 (48.8)
Divorced	6 (2.1)
No reply	5 (1.7)
Highest education:	
High school	126 (43.6)
Technical or junior college	42 (14.5)
Bachelor's degree	97 (33.6)
Master's degree or higher	23 (8.0)
No reply	1 (0.3)
Occupation:	
Employed	145 (50.2)
Housewife	54 (18.7)
Agriculture	1 (0.3)
Physical labor	6 (2.1)
Student or others	83 (28.7)

SD: standard deviation.

**Table 2:** Correlation coefficients and average variance extracted (AVE).

	Factor 1	Factor 2	Factor 3	Factor 4	Factor 5	Factor 6
Factor 1	(0.51)					
Factor 2	0.82	(0.52)				
Factor 3	0.87	0.81	(0.55)			
Factor 4	0.58	0.51	0.51	(0.62)		
Factor 5	0.55	0.61	0.71	0.46	(0.58)	
Factor 6	0.69	0.70	0.73	0.61	0.74	(0.42)

(AVE): average variance extracted.

**Table 3:** Factor loadings results from exploratory factor analysis.

Item	Factor loading				
	Factor 1	Factor 2	Factor 3	Factor 4	Factor 5
Neuropsychologic signs and symptoms					
(5) I feel my heart palpitate.	**0.79**	0.15	0.19	0.07	0.09
(7) I feel heavy in the chest.	**0.70**	0.18	0.17	0.33	0.15
(25) I have flank pain.	**0.58**	0.13	0.22	0.33	0.13
(6) I am startled by faint noise.	**0.57**	0.25	0.13	0.23	0.19
(4) I have ringing in the ears.	**0.53**	0.13	0.14	0.07	0.09
(3) I feel dizzy.	**0.53**	0.26	0.20	0.21	0.37
(11) I feel short of breath.	**0.51**	0.24	0.38	0.24	0.05
(14) I have a poor appetite.	**0.45**	0.24	0.20	0.28	0.14
(24) I have pain in the joints.	**0.43**	0.31	0.17	0.11	0.11
^*∗*^(20) I have a lump somewhere on my body.	0.30	0.18	0.19	0.07	0.16
Dermatologic and fatigue-related signs and symptoms					
(22) I have dark circles under the eyes.	0.07	**0.70**	0.15	0.16	0.10
(21) My face is yellowish.	0.23	**0.66**	0.21	0.05	0.06
(12) I feel fatigued.	**0.41**	**0.53**	0.08	0.21	0.19
(13) I feel heavy or weak in the limbs.	**0.51**	**0.52**	0.24	0.19	0.11
(18) My stomach or intestine rumbles.	0.28	**0.45**	0.03	0.23	0.07
^*∗*^(23) I feel itchy.	0.16	0.39	0.24	0.20	0.16
Respiratory signs and symptoms					
(9) I have sputum in my throat.	0.19	0.12	**0.81**	0.12	0.00
(8) I have a cough.	0.24	0.16	**0.71**	0.08	0.15
(10) I feel a foreign body present in the throat, neither swallowed nor ejected.	0.18	0.15	**0.63**	0.10	0.04
^*∗*^(19) My stool is mucousy.	0.10	0.28	0.29	0.14	0.06
Digestive signs and symptoms					
(16) I have indigestion.	0.19	0.23	0.15	**0.76**	0.18
(17) I have a feeling of fullness in the stomach with just a little food.	0.33	0.27	0.13	**0.70**	0.04
(15) I feel sick in the stomach.	0.35	0.17	0.27	**0.41**	0.17
Head-related signs and symptoms					
(2) I have a headache.	0.31	0.20	0.09	0.16	**0.90**
(1) I feel unclear in the head.	0.38	**0.40**	0.10	0.25	**0.42**
Rotation Sums of Squared Loadings					
% of variance	17.21	11.08	9.62	8.13	5.78
Cumulative%	17.21	28.29	37.90	46.03	51.81

Factors were extracted by maximum likelihood method and rotated by varimax rotation.

Bold values indicate factor loading of greater than 0.40.

^*∗*^Items whose loadings are less than 0.4 for every factor.

**Table 4:** Comparison of factor structure between this and a previous study.

Factor structure of this study		Factor structure of Park et al.'s study [4]	
Factor 1	Palpitation	Palpitation	Factor 1
	Startled by faint noise	Startled by faint noise	
	Feeling heavy in the chest	Feeling heavy in the chest	
	Joint pain	Joint pain	
	Shortness of breath	Shortness of breath	
	Tinnitus	Tinnitus	
	Flank pain	Flank pain	
	Dizziness		
	Poor appetite		
	Lumps		
Factor 4	Indigestion	Indigestion	Factor 2
	Feeling of abdominal fullness	Feeling of abdominal fullness	
	Sickness	Sickness	
		Poor appetite	
		Rumbling sound	
Factor 5	Headache	Headache	Factor 3
	Unclearness in the head	Unclearness in the head	
		Dizziness	
Factor 2	Fatigue	Fatigue	
	Feeling heavy in the limbs	Feeling heavy in the limbs	
	Dark circle under the eyes	Dark circle under the eyes	Factor 4
	Yellowish face	Yellowish face	
	Rumbling sound		
	Itching		
Factor 3	Sputum	Sputum	Factor 5
	Feeling of foreign body in the throat	Feeling of foreign body in the throat	
	Cough	Cough	
	Mucousy stool		
		Lumps	Factor 6
		Itching	
		Mucousy stool	

Items are shortened and reordered for easier comparison.

**Table 5:** Internal consistency of factors.

Cronbach's *α*	item	Cronbach's *α* if item is deleted
Factor 1 0.881	(5) I feel my heart palpitate.	0.860
	(7) I feel heavy in the chest.	0.858
	(25) I have flank pain.	0.863
	(6) I am startled by faint noise.	0.867
	(4) I have ringing in the ears.	0.876
	(3) I feel dizzy.	0.864
	(11) I feel short of breath.	0.869
	(14) I have a poor appetite.	0.873
	(24) I have pain in the joints.	0.877
	^*∗*^(20) I have a lump somewhere on my body.	0.882

Factor 2 0.811	(22) I have dark circles under the eyes.	0.774
	(21) My face is yellowish.	0.773
	(12) I feel fatigued.	0.770
	(13) I feel heavy or weak in the limbs.	0.761
	(18) My stomach or intestine rumbles.	0.799
	(23) I feel itchy.	0.809

Factor 3 0.76	(9) I have sputum in my throat.	0.632
	(8) I have a cough.	0.650
	(10) I feel a foreign body present in the throat, neither swallowed nor ejected.	0.689
	^*∗*^(19) My stool is mucousy.	0.808

Factor 4 0.786	(16) I have indigestion.	0.634
	(17) I have a feeling of fullness in the stomach with just a little food.	0.657
	^*∗*^(15) I feel sick in the stomach.	0.806

Factor 5 0.765	(2) I have a headache.	—
	(1) I feel unclear in the head.	—

^*∗*^Cronbach's *α* increases if item is deleted

— Cronbach's *α* cannot be calculated because factor 5 has only 2 items.

**Table 6:** 

Condition	1	2	3	4	5	6	7
(1) I feel unclear in the head.							
(2) I have a headache.							
(3) I feel dizzy.							
(4) I have ringing in the ears.							
(5) I feel my heart palpitate.							
(6) I am startled by faint noise.							
(7) I feel heavy in the chest.							
(8) I have a cough.							
(9) I have sputum in my throat.							
(10) I feel a foreign body present in the throat, neither swallowed nor ejected.							
(11) I feel short of breath.							
(12) I feel fatigued.							
(13) I feel heavy or weak in the limbs.							
(14) I have a poor appetite.							
(15) I feel sick to the stomach.							
(16) I have indigestion.							
(17) I have a feeling of fullness in the stomach with just a little food.							
(18) My stomach or intestine rumbles.							
(19) My stool is mucousy.							
(20) I have a lump somewhere on my body.							
(21) My face is yellowish.							
(22) I have dark circles under the eyes.							
(23) I feel itchy.							
(24) I have pain in the joints.							
(25) I have flank pain.							

1: disagree very strongly, 2: disagree strongly, 3: disagree, 4: neither agree nor disagree, 5: agree, 6: agree strongly, and 7: agree very strongly.

## Appendix

### Phlegm Pattern Questionnaire

We would like to know more about any problems you have experienced recently. Please answer all the questions by checking the answer that applies to you most closely.

See Table 6.
